# Examination of the Role of Mitochondrial Morphology and Function in the Cardioprotective Effect of Sodium Nitrite Administered 24 h Before Ischemia/Reperfusion Injury

**DOI:** 10.3389/fphar.2018.00286

**Published:** 2018-03-28

**Authors:** Vivien Demeter-Haludka, Mária Kovács, Alexandra Petrus, Roland Patai, Danina M. Muntean, László Siklós, Ágnes Végh

**Affiliations:** ^1^Department of Pharmacology and Pharmacotherapy, Albert-Szent Györgyi Medical Centre, University of Szeged, Szeged, Hungary; ^2^Department of Pathophysiology, “Victor Babes” University of Medicine and Pharmacy, Timisoara, Romania; ^3^Department of Biophysics, Biological Research Centre, Hungarian Academy of Sciences, Szeged, Hungary

**Keywords:** ischaemia/reperfusion, arrhythmia, sodium nitrite, cardioprotection, mitochondrial structure, mitochondrial respiration

## Abstract

**Background:** We have previous evidence that in anesthetized dogs the inorganic sodium nitrite protects against the severe ventricular arrhythmias, resulting from coronary artery occlusion and reperfusion, when administered 24 h before. The present study aimed to examine, whether in this effect changes in mitochondrial morphology and function would play a role.

**Methods:** Thirty dogs were infused intravenously either with saline (*n* = 15) or sodium nitrite (0.2 μmol/kg/min; *n* = 15) for 20 min, and 24 h later, 10 dogs from each group were subjected to a 25 min period of occlusion and then reperfusion of the left anterior descending coronary artery. The severity of ischaemia and ventricular arrhythmias were examined *in situ*. Left ventricular tissue samples were collected either before the occlusion (5 saline and 5 nitrite treated dogs) or, in dogs subjected to occlusion, 2 min after reperfusion. Changes in mitochondrial morphology, in complex I and complex II-dependent oxidative phosphorylation (OXPHOS), in ATP, superoxide, and peroxynitrite productions were determined.

**Results:** The administration of sodium nitrite 24 h before ischemia/reperfusion significantly attenuated the severity of ischaemia, and markedly reduced the number and incidence of ventricular arrhythmias. Nitrite also attenuated the ischaemia and reperfusion (I/R)-induced structural alterations, such as reductions in mitochondrial area, perimeter, and Feret diameter, as well as the increase in mitochondrial roundness. The administration of nitrite, however, enhanced the I/R-induced reduction in the mitochondrial respiratory parameters; compared to the controls, 24 h after the infusion of nitrite, there were further significant decreases, e.g., in the complex I-dependent OXPHOS (by −20 vs. −53%), respiratory control ratio (by −14 vs. −61%) and in the P/E control coupling ratio (by 2 vs. −36%). Nitrite also significantly reduced the I/R-induced generation of superoxide, without substantially influencing the ATP production.

**Conclusions:** The results suggest that sodium nitrite may have an effect on the mitochondria; it preserves the mitochondrial structure and modifies the mitochondrial function, when administered 24 h prior to I/R. We propose that nitrite affects primary the phosphorylation system (indicated by the decreased P/E ratio), and the reduction in superoxide production would result from the subsequent suppression of the ROS producing complexes; an effect which may certainly contribute to the antiarrhythmic effect of nitrite.

## Introduction

We have previous evidence that the acute administration of sodium nitrite (0.2 μmol/kg/min; i.v.), protects against the ischaemia and reperfusion (I/R)-induced severe ventricular arrhythmias, in anesthetized dogs (Kovács et al., 2015). This protection was associated with protein S-nitrosylation (SNO) and glutathionylation by nitric oxide (NO) derived from nitrite (Kovács et al., 2015). More recently, we have reported that sodium nitrite, administered 24 h prior to a similar period of I/R, evokes also an antiarrhythmic effect (Demeter-Haludka et al., 2017). This particular study has also examined whether this post-poned effect of nitrite against arrhythmias involves the mechanism of the nitric oxide (NO)-induced iNOS activation, which is known to play a significant role in the preconditioning-induced delayed cardioprotection (Végh and Parratt, 1996; Bolli et al., 1997). We have found that, in contrast to preconditioning, where the pharmacological inhibition of iNOS by S-(2-aminoethyl)-isothiourea completely abolished the delayed antiarrhythmic protection (Kis et al., 1999a,b; Babai et al., 2002), the nitrite-induced effect was only partially diminished following iNOS inhibition (Demeter-Haludka et al., 2017). This finding suggested that the nitrite-induced cardioprotective effect that occur 24 h after nitrite administration may involve additional mechanisms, which are most probably independent from the activation of iNOS (Demeter-Haludka et al., 2017).

There is some previous evidence for the late occurring cardioprotective effect of sodium nitrite in various *in vivo* and *in vitro* models of ischaemia and reperfusion (Shiva et al., 2007a,b; Shiva and Gladwin, 2009). For example, it has been found that sodium nitrite administered in rats, 24 h prior to I/R, reduced myocardial infarct size and hepatic reperfusion injury (Shiva et al., 2007a). This protection was attributed to a stable post-translational modification of the mitochondrial complexes (particularly complex I) via S-nitrosylation (Shiva et al., 2007b). Since there were no changes in mitochondrial respiration and ATP generation of the hepatic mitochondria, isolated from the nitrite treated rats until subjected them to anoxia and re-oxygenation (Shiva et al., 2007a), it was concluded that the rapid and prolonged S-nitrosylation of mitochondrial proteins, plays an important role in the delayed protective effect of nitrite (Shiva and Gladwin, 2009).

Starting from the assumption that the target of the cardioprotective effect of nitrite might be a mitochondria-mediated process, we designed studies, in which changes in mitochondrial morphology and in respiratory function were examined in dogs undergoing a 25 min period of coronary artery occlusion and reperfusion, 24 h after the administration of sodium nitrite.

## Materials and methods

### Ethics

The upkeep of the dogs was in accordance with Hungarian law (XVIII/VI/31) regarding large experimental animals, which conforms to the Guide for the Care and Use of Laboratory Animals by the US National Institutes of Health (NIH publication No.85-23, revised in 1996), and conformed to the European Parliament Directive 2010/63/EU. All animal experiments were supervised and approved by the Department of Animal Health and Food Control of the Ministry of Agriculture and Rural Development (No.XIII/1211/2012) and the Ethical Committee for the Protection of Animals in Research of University of Szeged, Szeged, Hungary (No.XIII./4657/2016).

### Surgical procedures

Thirty adult mongrel dogs of either sexwith a mean body weight of 22 ± 4 kg were used. The animals were housed in a separated animal room (temperature: 10–20°C, humidity: 40–70%, lightening: 12 h per day, 2 animals per pen) for 2 weeks and fed a standard diet and *ad libitum* access to water. Food was withdrawn 24 h before anesthesia. The surgical interventions were as the same as described previously (Végh et al., 1992; Demeter-Haludka et al., 2017). In brief, on day one, the dogs were lightly anesthetized with intravenous sodium pentobarbitone (30 mg/kg; Euthasol 40%, Produlab Pharma B.V., Netherlands), and a polyethylene catheter was introduced into the jugular vein for the administration of saline and sodium nitrite. A Millar tip catheter (5F, Millar Instruments Inc., USA) was also positioned into the left carotid artery to measure changes in arterial blood pressure. Twenty-four hours later (on day 2), the dogs were re-anesthetized with a bolus injection of sodium pentobarbitone (30 mg/kg, i.v.), and the anesthesia was maintained with intravenous injections of a mixture of chloralose and urethane (60 and 200 mg/kg respectively; Sigma, USA). The depth of anesthesia was monitored, and when it was necessary, a further bolus injection of the anesthetic was given. The dogs were ventilated with room air using a Harvard respirator (Harvard Apparatus, USA) at a rate and volume sufficient to maintain arterial blood gases within normal limits (Végh et al., 1992). Body temperature was measured from the mid-esophagus and maintained at 37 ± 0.5°C.

A Cordis F4 catheter was introduced into the right femoral artery to measure arterial blood pressure, whereas the Millar tip catheter, introduced previously into the left carotid artery, was pushed into the left ventricle (LV) to measure LV systolic and end-diastolic (LVEDP) pressure, as well as the LV positive and negative dP/dt_max_. After thoracotomy, the left anterior descending (LAD) coronary artery was prepared for occlusion proximal to the first main diagonal branch. Myocardial ischaemia was induced by a 25 min period of LAD occlusion, followed by 2 min reperfusion (Végh et al., 1992). The severity of ischaemia was assessed by measuring changes in the degree of inhomogeneity of electrical activation (expressed in milliseconds) and in the epicardial ST-segment (expressed in mV), using a composite electrode positioned within the ischaemic area (Végh et al., 1992; Demeter-Haludka et al., 2017). A chest lead II standard electrocardiogram was recorded to measure heart rate (HR) and to assess the severity of arrhythmias, such as the total number of ventricular premature beats (VPBs), the incidence and the number of episodes of ventricular tachycardia (VT), the incidence of ventricular fibrillation (VF) during occlusion, and the incidence of VF following reperfusion (Végh et al., 1992). Dogs that were still alive 2 min after reperfusion were considered to be survivors. These dogs were euthanized by an excess dose of the anesthetic 2 min after reperfusion. All parameters were recorded (Plugsys Hemodynamic Apparatus; Hugo Sachs Electronik, Germany), stored and evaluated by LabChart 7 (AD Instruments, Australia) software.

### *In vitro* measurements

#### Assessment of mitochondrial morphology

This was performed by transmission electron-microscopy (TEM). Blocks of fresh tissue samples (1 mm^3^), excised from the ischaemic region, were fixed in Karnovsky solution (Karnovsky, 1965) for 240 min at room temperature, rinsed and post-fixed in 2% OsO_4_ (Millonig, 1961). After dehydration with ethanol, the samples were embedded in epoxy resin (Durcupan ACM, Sigma, USA) and polymerized at 56°C for 2 days. Ultrathin sections (50 nm) were prepared and contrasted with uranyl acetate (Hayat, 1970) and lead citrate (Reynolds, 1963). Transmission electron-microscope (Zeiss CEM 902, Germany) was used in conventional transmission mode (80 keV) to capture sub-sarcolemmal (SSM), perinuclear (PN) and inter-myofibrillar (IMF) mitochondria, using a Spot RT 14.0 CCD camera (Diagnostic Instruments, USA) at 12,000 x magnifications. Five images were taken from each area per samples, and the mitochondria were segmented with ImageJ 2 (FIJI; NIH, Bethesda, USA). Changes in mitochondrial morphology were evaluated using the built-in applications of ImageJ 2; such as we determined the area (μm^2^) and perimeter (μm), the measures of the size of the mitochondria, as well as the roundness (4x[Area]/(πx[Major axis]^2^) and the Feret diameter (μm), which describe the level of circularity and the shape of the mitochondria. Data obtained from the five images in each animal were averaged, and the results obtained from the individual dogs within a certain group were also averaged. These values served for comparison among the groups.

#### Assessment of mitochondrial respiration and ATP production

Mitochondrial respiration was measured by Clarke-type oxygen electrode (Strathkelvin 782 oxygen system, Strathkelvin, Germany). Tissue samples collected from the ischaemic area was homogenized in isolation medium (Grainer, Strathkelvin, Germany), containing trypsin and sucrose, and the mitochondria were separated by centrifugation. The concentrations of the mitochondrial proteins were determined by the method of Bradford.

The respiratory parameters for CI and CII were determined as described previously (Duicu et al., 2013a,b). We measured the basal respiration (State 2), the active respiration (OXPHOS; State 3), the capacity of the inhibition of OXPHOS (State 4; oligomycin, 2 μM, Sigma, USA) and the electron transport system (ETS). The intactness of the outer mitochondrial membrane (Pc) was evaluated by the administration of 10 μM cytochrome C (Sigma, USA). The uncoupling was determined using carbonyl-cyanide-p-(trifluoro-methoxy) phenyl-hydrazone (FCCP; 0.5 μM, Sigma USA). Antimycin A (Sigma, USA) was administered to assess the residual oxygen consumption. From the measured parameters the respiratory control ratio (RCR = OXPHOS/State4) and the P/E coupling control ratio (OXPHOS/ETS) were calculated. The measurements were repeated three times in each sample per dog, and the results were averaged. Data obtained from the individual dogs within a group were also averaged, and these means served for the comparison among the groups.

Mitochondrial ATP production was assessed by bioluminescence assay, using an ATP Determination Kit (Invitrogen, USA) according to the manufacturer's protocol. Malate and pyruvate (Sigma, USA) were used as substrates. The emitted light was measured with luminescent optic using a micro plate reader (FLUOstar OPTIMA, Germany). Data were expressed as relative luminescence units (RLU). Three samples in each dog were evaluated and then averaged within a certain group. These means were compared among the groups.

#### Assessment of tissue superoxide production

Superoxide production was determined as described previously (Kiss et al., 2010). The preparation of tissue samples, collected from the ischaemic and non-ischaemic areas within 2 min of the reperfusion. Longitudinal cryosections (20 μm) were cut, stained with dihydroethidium (DHE, 10 μM, Sigma, USA). N-acetyl-L-cysteine (100 mM, Sigma, USA) was used as a negative control. Both from the stained and negative control samples, ten images were captured by a confocal laser scanning microscope (Olympus FV 1000, Japan). The intensity of the fluorescent signals was analyzed by ImageJ, and expressed in arbitrary units. The intensity values, evaluated from four images in each dogs, were averaged, and data obtained from dogs within a certain group were also averaged. These values served for comparison among the groups.

#### Assessment of peroxynitrite production

This was assessed by measuring 3-nitrotyrosine (3-NT) formation using Western blot. Tissue samples (70 mg), taken from the ischaemic myocardium within 2 min of reperfusion, were prepared as described previously (Kiss et al., 2010). The formation of 3-NT was assessed from 25 μg of total protein loaded onto SDS-PAGE gel (10%) and transferred to PVDF membrane. Mouse monoclonal anti-nitrotyrosine was used as primary antibody (diluted to 1:3000; Chemicon, Millipore, USA), and horseradish peroxidase-conjugated rabbit anti-mouse IgG (diluted to 1:1000, Dakocytomation, Denmark) was used as a secondary antibody. The blot was developed with an enhanced chemiluminescence kit (ECL Plus, GE Healthcare, UK), exposed to X-ray film and scanned. The intensity of the 3-NT bands was determined using Image J software, and expressed in percentage of the sham-operated animals. Equal loading of the samples was controlled by Coomassie Brilliant Blue staining, and normalized for total protein. Protein samples, isolated from four dogs in each experimental group, were used for western blot. The measurements were repeated three times in each dog, and the results were averaged. Data obtained from the individual dogs within a group were also averaged, and these means served for the comparison among the groups.

### Experimental protocol

Thirty dogs of both sexes were randomly divided into four groups. On day one, 15 dogs (7 female and 8 male) were infused intravenously with saline, and another 15 dogs (6 female and 9 male) with sodium nitrite (0.2 μmol/kg/min) for 20 min. Twenty-four hours later, 10 control (IC) and 10 nitrite (NaNO_2_+I/R) treated dogs underwent a 25 min period of LAD occlusion followed by rapid reperfusion. In 5 nitrite (NaNO_2_) and in 5 saline (SC) treated dogs (both groups contained 2 female and 3 male, undergoing the same surgical interventions, without subjecting them to I/R), the hearts were removed 24 h after nitrite and saline administration, respectively.

At the end of the experiments, the hearts were stopped by an excess of anesthetic, removed and myocardial tissue samples were taken for *in vitro* analyses. In dogs that were fibrillated on reperfusion, the samples were collected at the time of the fibrillation observed. The samples were either immediately used (for the mitochondrial measurements) or frozen in liquid nitrogen and stored on −80°C. In 4 or 5 dogs from the IC and NaNO_2_+I/R groups, the “risk area” was assessed using Patent Blue V dye, as described previously (Végh et al., 1992; Demeter-Haludka et al., 2017).

### Statistical analysis

The data were expressed as mean ± SEM, and differences between means were compared by Welch-ANOVA for repeated measures the Bonferroni-Holm *post-hoc* test. The number of VPBs and the number of episodes of VT were compared using the Kruskal-Wallis test. The incidence of VT and VF, as well as survival from the combined I/R insult was compared by the Fisher Exact test. Differences between groups were considered significant at *P* < 0.05.

## Results

### Haemodynamic changes following nitrite administration and coronary artery occlusion

The intravenous infusion of sodium nitrite significantly reduced the mean arterial blood pressure from 132 ± 5 to 122 ± 6 mmHg (*P* < 0.05), without a substantial increase in the heart rate (from 167 ± 7 to 168 ± 11 beats/min). Twenty-four hours later, when the dogs had been subjected to a 25 min period of occlusion, there were similar changes in most of the haemodynamic parameters, except that the increase in LVEDP and the decrease in negative dP/dt_max_ were significantly less in the nitrite than in the saline infused dogs (Table 1).

**Table 1:** Haemodynamic changes during a 25 min occlusion of the LAD.

	**Saline**		**NaNO**_**2**_	
	**Baseline**	**Max. change**	**Baseline**	**Max. change**
SABP (mmHg)	145 ± 14	−17 ± 3^*^	141 ± 4	−10 ± 5^*^
DABP (mmHg)	101 ± 10	−18 ± 3^*^	97 ± 4	−11 ± 6^*^
MABP (mmHg)	116 ± 11	−17 ± 2^*^	111 ± 3	−11 ± 5^*^
LVSP (mmHg)	139 ± 11	−25 ± 5^*^	143 ± 13	−9 ± 6^*^
LVEDP (mmHg)	6.6 ± 1.0	7.1 ± 1.4^*^	4.4 ± 1.6	5.4 ± 0.7^*^^#^
+dP/dt_max_(mmHg/s)	2869 ± 226	−769 ± 78^*^	2839 ± 138	−557 ± 148^*^
−dP/dt_max_ (mmHg/s)	2609 ± 163	−574 ± 193^*^	2295 ± 63	−147 ± 118^*^^#^
HR (beats/min)	168 ± 7	5 ± 5	165 ± 8	−5 ± 2

*Mean ± SEM, calculated from n = 10 experiments*.

* *P < 0.05 vs. baseline value*,

# *P < 0.05 vs. saline treated control group*.

*SABP, systolic arterial blood pressure; DABP, diastolic arterial blood pressure; MABP, mean arterial blood pressure; LVSP, left ventricular systolic pressure; LVEDP, left ventricular end-diastolic pressure; HR, heart rate*.

### The administration of sodium nitrite reduces the number and incidence of ventricular arrhythmias during coronary artery occlusion and reperfusion

**Figure 1:**
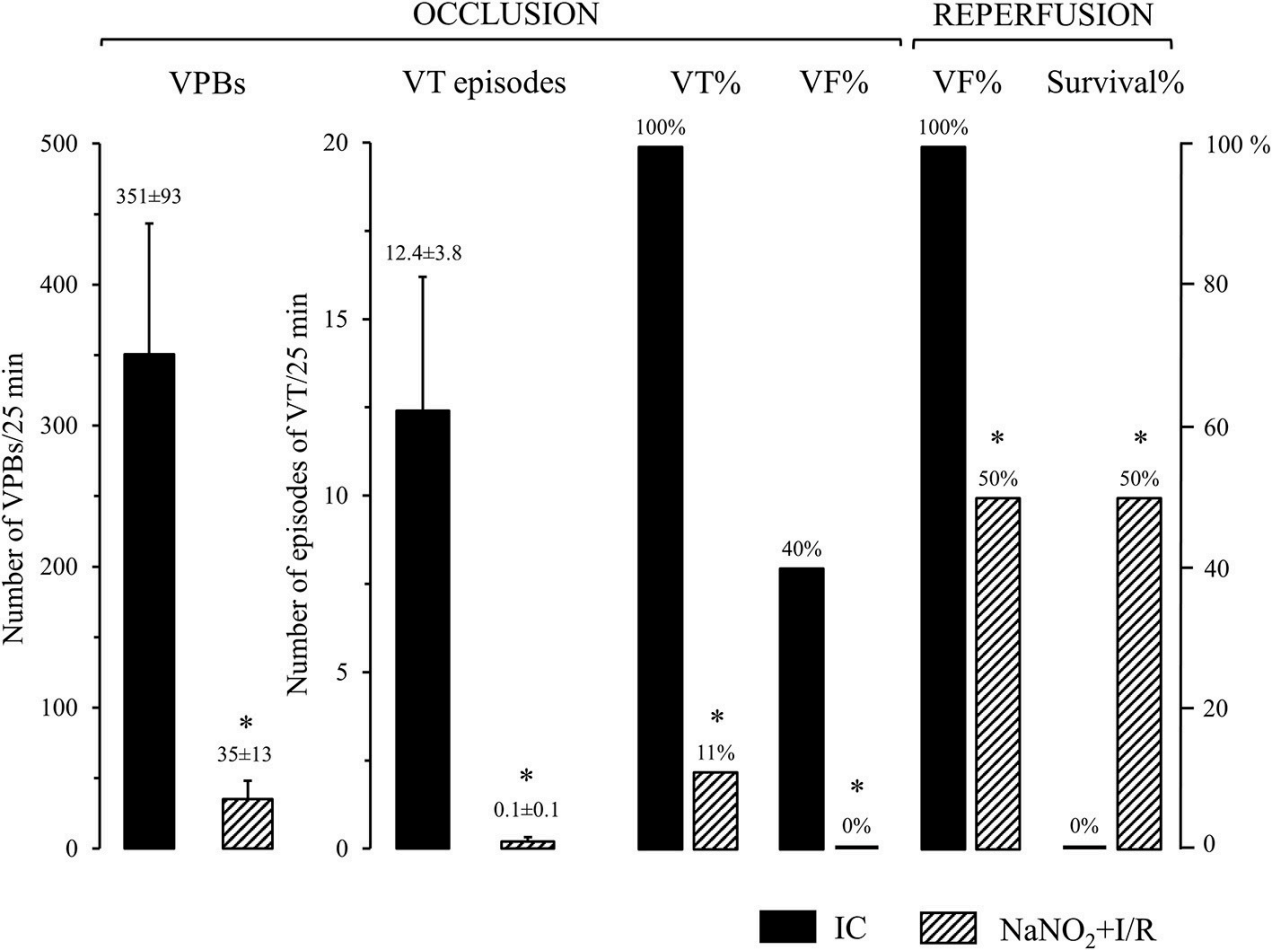
The severity of arrhythmias during a 25 min occlusion and reperfusion of the LAD in control dogs (IC; *n* = 10) and in dogs infused with sodium nitrite (NaNO_2_+I/R; *n* = 10), 24 h previously. Sodium nitrite markedly reduced the total number of ventricular premature beats (VPBs), the incidence and the number of episodes of ventricular tachycardia (VT), the incidence of ventricular fibrillation (VF) during occlusion, and increased survival from the combined ischaemia and reperfusion insult. Values are means ± SEM. ^*^P < 0.05 compared with ischaemic controls.

This is illustrated in Figure 1. Control dogs, showed a great number of VPBs and episodes of VT that occurred in all dogs (100%) during the 25 min LAD occlusion. Further, four animals out of the 10 (40%) fibrillated during the occlusion and all the remaining dogs fibrillated on reperfusion; thus no control dog survived the combined I/R insult. In contrast, dogs infused with sodium nitrite 24 h previously, exhibited significantly less number of VPBs and episodes of VT that occurred in only 1 dog (10%) during the occlusion period. Moreover, no dog in the nitrite treated group fibrillated during the occlusion and 50% of the dogs survived reperfusion.

### The administration of sodium nitrite attenuates the severity of ischaemia during coronary artery occlusion

This was assessed by measuring changes in the epicardial ST-segment and the degree of inhomogeneity of electrical activation during a 25 min occlusion of the LAD as described previously (Végh et al., 1992). In control dogs both indices of ischaemia severity were steeply increased, reaching the maximum value (epicardial ST segment: 9.3 ± 0.9 mV, degree of inhomogeneity: 125 ± 12 mV) by the 5 min of the occlusion, and these were maintained over the rest of the occlusion. The administration of nitrite significantly attenuated these ischaemia-induced changes in the epicardial segment (3.7 ± 0.6 mV) and inhomogeneity (63 ± 13 ms) during the entire occlusion period, although there were no significant differences between the groups, regarding the risk area (39.2 ± 1.2 vs. 40.3 ± 1.2 in the control and in the nitrite group, respectively).

### The administration of sodium nitrite reduces the ischaemia and reperfusion-induced morphological changes of the mitochondria

**Figure 2:**
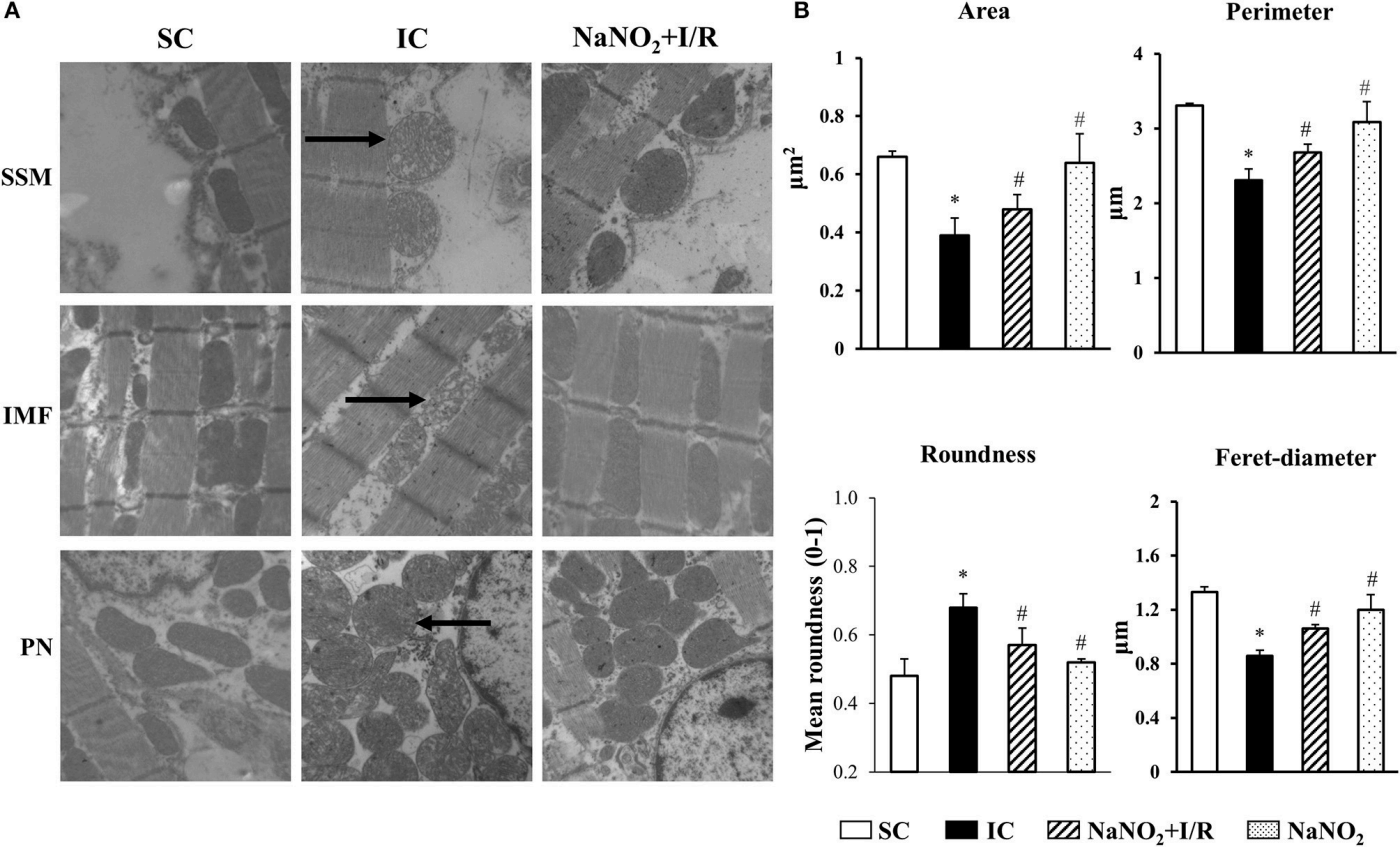
Representative images **(A)** and the quantitative analysis **(B)** acquired by transmission electronmicroscopy (TEM) in sham-operated control (SC; *n* = 4), ischaemic control dogs (IC; *n* = 6), and in dogs infused with nitrite with (NaNO_2_+I/R; *n* = 4) and without ischaemia (NaNO_2_; *n* = 4). For each *n*, 5 images were evaluated and averaged. **(A)** There were no significant differences between the sub-sarcolemmal (SSM), inter-myofibrillar (IMF) and the perinuclear (PN) regions, concerning the changes of mitochondrial morphology in response to I/R or nitrite treatment. The images show that compared to the SC group, in dogs subjected to a 25 min I/R, substantial swelling and disorganization of cristae of the mitochondrial matrix occurred in all the three examined regions (indicated by arrows). These alterations were less marked in dogs treated with sodium nitrite, 24 h previously. **(B)** This shown data obtained from mitochondria positioned in the IMF region. Compared to the SC dogs, the mitochondrial area, perimeter and Feret-diameter were significantly reduced, whereas the mitochondrial roundness was markedly increased in the IC dogs. These alterations were significantly less marked following the administration of sodium nitrite. Values are means ± S.E.M. ^*^P < 0.05 compared with SC; ^#^*P* < 0.05 compared with IC.

The representative images acquired by TEM are illustrated in Figure 2A, whereas data of the quantitative analysis obtained from mitochondria localized in the sub-sarcolemmal (SSM), inter-myofibrillar (IMF) and perinuclear (PN) areas, are summarized in Table 2, and the results of mitochondria, assessed in the IMF region, are also illustrated in Figure 2B. The images show that compared to the SC dogs, in dogs of the IC group a substantial swelling and disorganization of cristae of the mitochondrial matrix could be observed, irrespective of their localization (SSM, PN, and IMF). These I/R-induced alterations were less marked in dogs infused with sodium nitrite, 24 h previously (Figure 2A). Furthermore, there were slight, but statistically not significant structural differences between the mitochondria, assessed in the three subsets in the sham control dogs (Table 2). A 25 min I/R resulted in similar tendency of changes in all mitochondria; thus, compared to the SC dogs, in dogs subjected to I/R there were significant reductions in the mitochondrial area, perimeter, and Feret diameter, and a significant increase in mitochondrial roundness (Table 2, Figure 2B). These alterations were significantly less marked in the nitrite treated animals (Table 2, Figure 2B). Sodium nitrite itself without ischaemia did not cause significant alterations in the assessed morphological parameters (Table 2, Figure 2B).

**Table 2:** Morphological changes in the different mitochondria subsets following ischaemia and reperfusion, and sodium nitrite administration.

	**Area (μm^2^)**	**Perimeter (μm)**	**Feret diameter (μm)**	**Roundness**
**SC (*****n*** = **4)**				
SSM	0.48 ± 0.02	2.69 ± 0.03	1.04 ± 0.01	0.57 ± 0.05
IMF	0.68 ± 0.04	3.38 ± 0.06	1.37 ± 0.03	0.46 ± 0.03
PN	0.49 ± 0.02	2.77 ± 0.09	1.09 ± 0.04	0.58 ± 0.02
**IC (*****n*** = **6)**				
SSM	0.35 ± 0.02^*^	2.12 ± 0.05^*^	0.77 ± 0.02^*^	0.75 ± 0.01^*^
IMF	0.39 ± 0.04^*^	2.30 ± 0.08^*^	0.88 ± 0.03^*^	0.67 ± 0.03^*^
PN	0.39 ± 0.02^*^	2.26 ± 0.05^*^	0.82 ± 0.02^*^	0.75 ± 0.02^*^
**NaNO**_**2**_**-IC (*****n*** = **4)**				
SSM	0.65 ± 0.05^#^	3.15 ± 0.12^#^	1.22 ± 0.07^#^	0.58 ± 0.07^#^
IMF	0.54 ± 0.04^#^	2.88 ± 0.06^#^	1.13 ± 0.01^#^	0.53 ± 0.04^#^
PN	0.51 ± 0.02^#^	2.76 ± 0.05^#^	1.04 ± 0.02^#^	0.65 ± 0.02^#^
**NaNO**_**2**_ **(*****n*** = **4)**				
SSM	0.58 ± 0.06^#^	2.96 ± 0.15^#^	1.13 ± 0.05^#^	0.58 ± 0.02^#^
IMF	0.67 ± 0.08^#^	3.18 ± 0.19^#^	1.22 ± 0.03^#^	0.54 ± 0.01^#^
PN	0.47 ± 0.02^#^	2.64 ± 0.08^#^	1.02 ± 0.03^#^	0.60 ± 0.02^#^

*For each n, 5 images were evaluated and averaged. Values are means ± S.E.M*.

* *P < 0.05 compared with SC*;

# *P < 0.05 compared with IC*.

*SSM, sub-sarcolemmal; IMF, inter-myofibrillar; PN, perinuclear*.

### The administration of sodium nitrite reduces mitochondrial respiration following coronary artery occlusion and reperfusion

**Figure 3:**
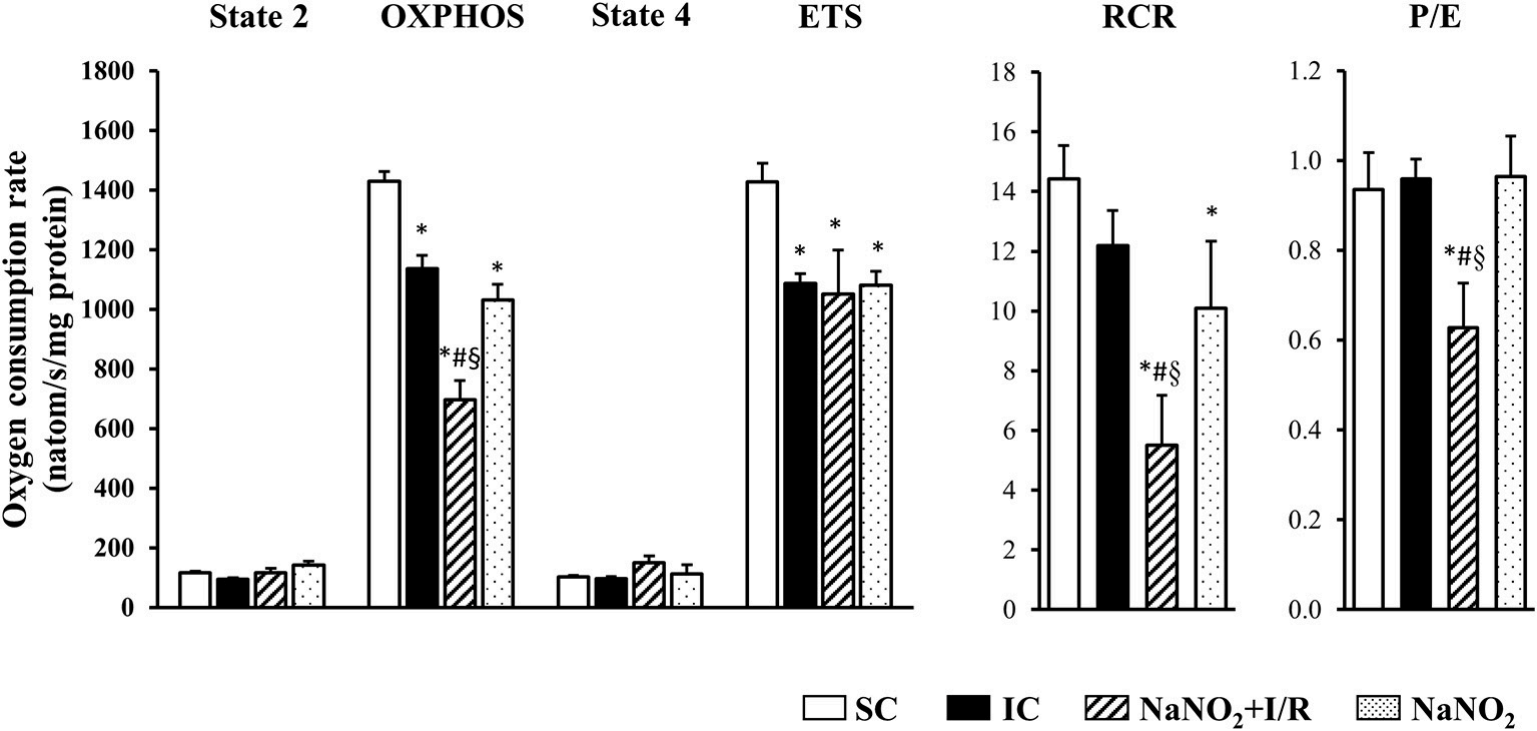
The effect of sodium nitrite administration on CI-dependent basal (State 2) respiration, on OXPHOS capacity and capacity of OXPHOS inhibition (State 4), as well as on the respiratory control ratio (RCR), the uncoupling of ETS and the P/E coupling ratio. There were no significant differences in the basal respiration between the examined groups. In dogs subjected to ischaemia and reperfusion (I/R), the CI-dependent OXPHOS and the ETS were significantly reduced, whereas the RCR was also, but not significantly, decreased. In these dogs the P/E coupling ratio did not differ from the SC dogs. Nitrite itself significantly reduced OXPHOS, ETS and RCR, without substantially modifying State 4 respiration and the P/E coupling ratio, compared with the SC dogs. In the nitrite treated dogs, subjected to I/R, there were further significant decreases in OXPHOS and RCR, and an increase in State 4 respiration, compared with the IC dogs. Since in these dogs ETS slightly increased compared with the IC dogs, the P/E coupling ratio was markedly reduced. Values are means ± S.E.M. from *n* = 5 animals/group. Values for each *n* were calculated from three replicates. ^*^P < 0.05 compared with SC; ^#^*P* < 0.05 compared with IC, ^§^*P* < 0.05 compared with nitrite alone.

**Figure 4:**
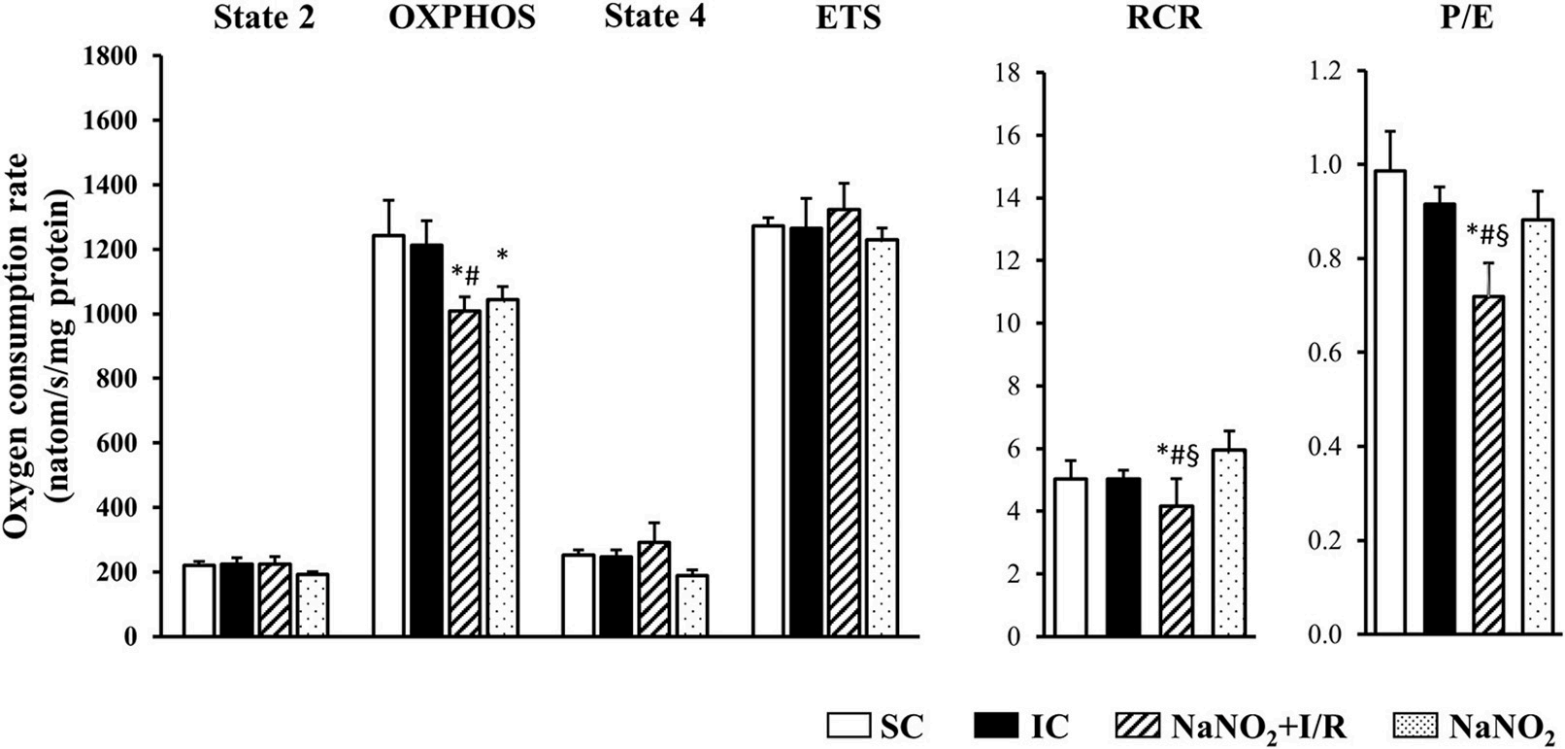
The effect of sodium nitrite on CII-dependent basal (State 2) respiration, on OXPHOS capacity and capacity of OXPHOS inhibition (State 4), as well as on the respiratory control ratio (RCR), the uncoupling of ETS and the P/E coupling ratio. There were no significant differences between groups as regards the basal (State 2) respiration. In dogs subjected to ischaemia and reperfusion (I/R) no significant alterations occurred in the CII-dependent respiratory parameters. The administration of nitrite affected only the OXPHOS, which further reduced when the nitrite treated dogs had been subjected to I/R. In these dogs the CII-dependent RCR and P/E were also significantly decreased compared to the IC dogs. Values are means ± S.E.M. from *n* = 5 animals/group. Values for each *n* were calculated from three replicates.^*^P < 0.05 compared with SC; ^#^*P* < 0.05 compared with IC, ^§^*P* < 0.05 compared with nitrite alone.

The changes in the CI and CII-dependent respiratory parameters are illustrated in Figures 3, 4, respectively. Whereas, there was no significant difference in the basal respiration between the examined groups, the CI-dependent OXPHOS and the ETS were markedly reduced in dogs subjected to a 25 min period of occlusion and then reperfusion. The respiratory control ratio (RCR), a classical parameter for the mitochondrial qualitative control, indicating the coupling between oxygen consumption and oxidative phosphorylation (Montaigne et al., 2010) was only slightly, but not significantly reduced following such aperiod of I/R insult (Figure 3). Furthermore, the P/E control coupling ratio, a measure of the limitation of OXPHOS capacity by the phosphorylation system, was almost the same in the ischaemic (IC group) as in the non-ischaemic (SC group) dogs, regarding both the CI and the CII-dependent respiration (Figures 3, 4, respectively). Interestingly, compared to the SC dogs, nitrite alone (without I/R) significantly reduced the CI-dependent OXPHOS, ETS, and RCR, without substantially modifying State 4 and the P/E coupling ratio (Figure 3). Furthermore, in dogs infused with nitrite and 24 h later subjected to a 25 min period of ischaemia and reperfusion, significant decreases occurred both in CI and CII-dependent OXPHOS and RCR, and an increase in State 4, compared with the ischaemic controls (Figure 3, 4). Since, in these dogs the ETS was slightly but not significantly increased compared with the untreated ischaemic (IC) dogs, the P/E coupling ratio was markedly reduced (Figures 3, 4), indicating that under conditions of ischaemia and reperfusion, nitrite limits OXPHOS capacity by influencing the phosphorylation system.

### Changes in the mitochondrial ATP production 24 h after sodium nitrite administration

Changes in total ATP production were determined in three samples of each animal, collected from the sham control (SC; *n* = 4), ischaemic control (IC; *n* = 5) dogs, as well as from dogs that had been infused with nitrite with (NaNO_2_+I/R; *n* = 5) and without (NaNO_2_; *n* = 5) ischaemia. The production of ATP was expressed in RLU (over 30 s/mg protein). Compared with the SC group, a 25 min period of I/R almost halved the ATP production (12232 ± 1291 cp. 7213 ± 1117 RLU/30 s/mg protein; *P* < 0.05). The administration of nitrite alone (13001 ± 3109 RLU/30 s/mg protein cp. SC group), and under ischaemic conditions (7130 ± 1560 RLU/30 s/mg protein cp. IC group) did not significantly modify the rate of ATP production.

### Changes in the ischaemia and reperfusion-induced superoxide production 24 h after sodium nitrite infusion

**Figure 5:**
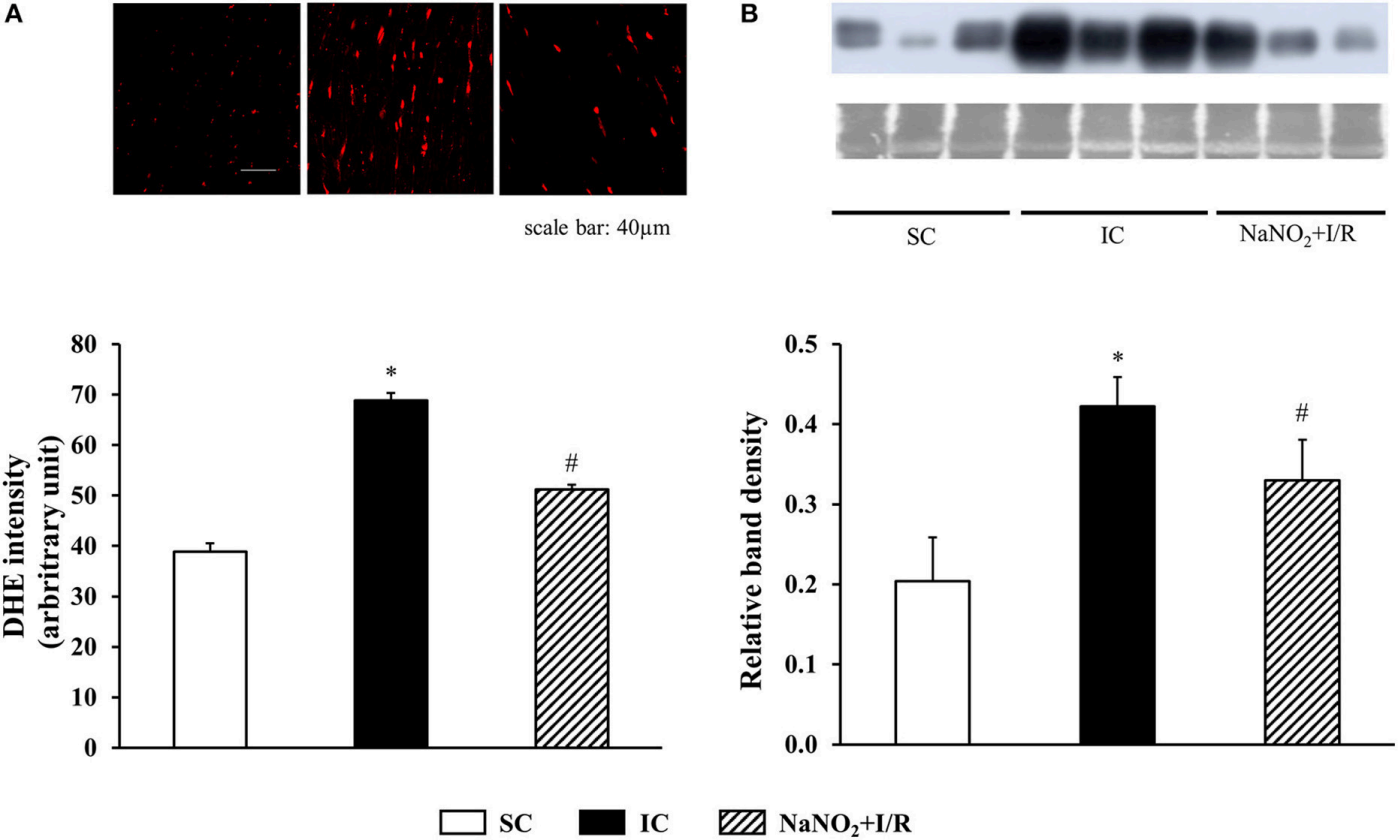
The effect of sodium nitrite administration on ischaemia and reperfusion-induced **(A)** superoxide and **(B)** 3-nitrotyrosine (3-NT) productions, The superoxide production was determined in 5 sham control (SC), ischaemic control (IC) and nitrite treated (NaNO_2_+I/R) dogs. For each *n*, 4 images were evaluated and averaged. The formation of 3-NT was assessed in four dogs in each group. Values for each *n* were calculated from three replicates. The representative images of the DHE staining and western blot with the corresponding Coomassie Blue staining are shown on the upper parts of the figure. Compared with the sham controls, occlusion and reperfusion of the LAD resulted in a marked increase in both the superoxide and 3-NT production. These changes were significantly less marked in dogs treated with nitrite 24 h previously and subjected to a similar period of I/R. Values are means ± S.E.M. ^*^P < 0.05 compared with SC; ^#^*P* < 0.05 compared with IC.

This is illustrated in Figure 5A. Compared to the SC dogs, the generation of superoxide was markedly increased in the IC dogs. This I/R-induced increase in superoxide production was attenuated by the prior administration of nitrite.

### Changes in the ischaemia and reperfusion-induced peroxynitrite production 24 h after the infusion of sodium nitrite

The changes in 3-NT production are shown in Figure 5B. Compared to the SC dogs, a 25 min I/R resulted in a significant increase in 3-NT production. This increase in 3-NT formation was markedly reduced in the nitrite treated dogs.

## Discussion

We have previous evidence that the infusion of sodium nitrite provides a marked immediate (Kovács et al., 2015), and also a later appearing (24 h later; Demeter-Haludka et al., 2017) protective effect against those severe ventricular arrhythmias that result from a 25 min period of coronary artery occlusion and reperfusion in anesthetized dogs. We have now examined, whether the cardioprotective effect of sodium nitrite, occurring 24 h later, involves changes in mitochondrial morphology and function. This question was raised because our previous studies, examining the role of NO-induced iNOS activation in this protection against arrhythmias showed that the NO/iNOS/NO pathway (Végh and Parratt, 1996; Bolli et al., 1997) may have some role in the protection, but it does not fully explain the marked antiarrhythmic effect of nitrite (Demeter-Haludka et al., 2017). Since, there has been some previous evidence, albeit from different experimental models, which suggests that the mitochondria might be important target organelles in the delayed protective effect of nitrite (Shiva et al., 2007a,b; Shiva and Gladwin, 2009), we designed studies to examine the effects of nitrite on mitochondrial structure and function in our established *in vivo* canine model of ischaemia and reperfusion (e.g., Végh et al., 1992; Kiss et al., 2010). Using various *in vitro* methods, we have determined the changes in mitochondrial morphology, the alterations in the CI and CII-dependent mitochondrial respiration, as well as in ATP, superoxide and peroxynitrite productions in myocardial tissue samples, collected from the heart of dogs during the early period (2 min) of reperfusion, following a 25 min ischaemic insult.

There is emerging evidence that changes in mitochondrial morphology play an important role both in the normal and the diseased myocardium; by the dynamic nature of the mitochondria their morphological changes may occur during cardiac development, and also in response to injurious conditions, such as ischaemia and reperfusion, heart failure, diabetes, apoptotic, and autophagy cell death (Ong and Hausenloy, 2010). In our study the qualitative and quantitative analyses of the TEM images showed that a 25 min period of ischaemia and 2 min reperfusion resulted in substantial structural alterations in the mitochondria, irrespective whether they were inter-myofibrillar, sub-sarcolemmal, or perinuclear mitochondria (Figure 2A).We have found that the electron density of the mitochondrial matrix was markedly reduced and the normally tightly packed cristae became disconnected and disorganized. There were also signs of mitochondrial swelling. In many of these severely damaged mitochondria, large and empty blebs could be observed that led to membrane disruption. In other mitochondria, a rearrangement of the cristae was apparent (Figure 2A). Furthermore, the reduction in the mitochondrial area, perimeter, and Feret-diameter, as well as the increase in roundness indicated that the mitochondria become smaller and more spherical following a 25 min period of ischaemia and reperfusion insult (Figure 2B). A recent finding also shows that in mouse subjected to a 20 min global ischaemia without reperfusion, the sphericity of the mitochondria, in all the three subsets, was significantly increased (Kalkhoran et al., 2017). We have also found that the I/R-induced structural changes of the mitochondria were significantly less marked, if the dogs had been infused with sodium nitrite, 24 h previously (Figure 2B). To the best of our knowledge, this is the first study, which has examined the effect of nitrite on mitochondrial morphology in a large animal model, and showed that nitrite may modify the ischaemia and reperfusion-induced structural changes of the mitochondria; an effect which might have a role in the cardioprotective effect of nitrite. However, as to whether nitrite directly acts on the mitochondria, or whether the preservation of mitochondrial morphology results from other effects of nitrite, we do not know; this warrants further examinations.

Also, we do not have direct evidence whether the preservation of mitochondrial structure by nitrite contributes to better mitochondrial function, but the results of the functional measurements show that nitrite modifies mitochondrial respiration and ROS production as well. Although there are many possibilities to assess mitochondrial function and dysfunction, in our experiments we measured mitochondrial respiration, as the generally accepted indicator of mitochondrial function (Brand and Nicholls, 2011) in isolated mitochondria, obtained from the control and the nitrite treated dog hearts. We have found that a 25 min ischaemia and 2 min reperfusion depressed mitochondrial respiration; i.e., both the CI and CII-dependent OXPHOS were significantly decreased, and there were also reductions in RCR (OXPHOS/state4) and in the ETS (Figures 3, 4). Since, the P/E control coupling ratio was similar in the ischaemic and in the non-ischaemic control groups, we suppose that the reduced mitochondrial respiration resulted primary from the depression of the respiratory complexes (mainly CI) of the ETS.

Interestingly, nitrite alone reduced the mitochondrial respiration 24 h later, and this was even further decreased, when the nitrite-treated dogs had been subjected to ischaemia and reperfusion. Thus, compared with the ischaemic controls (IC group), in the nitrite treated dogs both the CI and CII-dependent OXPHOS, the RCR, and the P/E coupling control ratio were significantly reduced. Furthermore, nitrite significantly reduced the superoxide and the 3-NT productions, resulted from a 25 min period of occlusion and reperfusion insult (Figure 5).

There is substantial evidence that NO regulates ROS formation, and that this mechanism is largely involved in the protective effect of NO, for example, against those severe ventricular arrhythmias (Kiss et al., 2010), which occur during the first minutes of the reperfusion, when the burst of ROS is apparent (Xia and Zweier, 1997; Iwase et al., 2007; Burwell and Brookes, 2008). There are, of course, a number of ways by which NO may regulate ROS formation. For example, NO inhibits the activities of xanthine/xanthine oxidase (Ichimori et al., 1999) and the NADPH oxidase (Clancy et al., 1992; Fujii et al., 1997), which are the major sources of ROS production. The other potential source of ROS is the mitochondrial respiratory chain, especially in the heart, where the myocytes are abundant in mitochondria. Thus, the mitochondrial electron transport might become an important sub-cellular source of ROS, and a contributor to the reperfusion-induced injury (Ambrosio et al., 1993). There is evidence that NO reduces mitochondrial superoxide production by acting directly on the ETS or the uncoupling proteins (Burwell and Brookes, 2008), but the precise mechanisms are still not clarified. Recently, it has been suggested that the redox-modification of specific cysteine-thiol groups of proteins in the subunits of the respiratory chain complexes with S-nitrosylation influences the respiratory chain activity, and modifies ROS production (Dröse et al., 2014). Indeed, the reversible S-nitrosylation of CI was protective against myocardial I/R damage (Couchani et al., 2013). Although in the present study we did not measure protein SNO, our previous results have revealed that following acute administration (just prior to ischaemia or reperfusion) nitrite protects the myocardium by S-nitrosylation, and perhaps by glutathionylation (Kovács et al., 2015). As to whether in our model SNO may play a role in the late antiarrhythmic effect of nitrite warrants further investigations.

It seems well accepted that CI and, especially in cardiac myocytes, complex III (CIII) are the main sources of superoxide production (Turrens, 2003), but more recently, CII has also been considered as an important generator of ROS, under certain circumstances (Turrens, 2003; Dröse et al., 2014). The contribution of these sites for the overall ROS production depends on the organ, the milieu of substrates and redox conditions, as well as on the intactness of the respiratory chain activity (St-Pierre et al., 2002; Turrens, 2003; Dröse et al., 2014). As the respiratory chain becomes reduced, such as during ischaemia and reperfusion or following a defect of mitochondrial complexes, electrons leak from the defective complex, resulting in the univalent reduction of oxygen to form superoxide. More recently, however, it is turned out that the inhibition of CI and CII activity attenuates the electron transfer to CIII, diminishes CIII reduction and decreases the electron leakage and the formation of ROS at CIII (Chen et al., 2003, 2006; Stewart et al., 2009), thereby protecting the myocardium against the reperfusion injury (Chen et al., 2006; Stewart et al., 2009).

In our dog model a 25 min ischaemia and 2 min reperfusion (this reperfusion interval was selected because the severe reperfusion-induced arrhythmias occur almost immediately after the reopening of the coronary artery; Figure 1) resulted in a mild, but significant reduction in the CI (24%; *P* < 0.05 compared to the SC group; Figure 3), and also in the CII-supported OXPHOS (Figure 4), a decrease in ATP and an increase in superoxide (Figure 5) productions. Furthermore, in these ischaemic dogs, the P/E coupling ratio was similar to that observed in the sham controls (SC), suggesting that such a period of I/R limits the capacity of the respiratory complexes of the ETS, and consequently, increases the generation of ROS. In contrast, the administration of nitrite itself (without I/R), and also following an occlusion and reperfusion insult, substantially reduced mitochondrial respiration; i.e., there was a marked decrease in the CI-dependent OXPHOS (48% compared with 24% in the IC group), in RCR and, in particular, in the P/E coupling ratio. The decrease in P/E following nitrite raises the possibility that nitrite (NO) affects the phosphorylation system, and that the reduction in the CI-dependent OXPHOS would result from the modification of the phosphorylation system rather than of the proximal complexes. Interestingly, despite the marked reduction in OXPHOS, the ATP production in the nitrite treated dogs was as the same as in the ischaemic, untreated controls. In contrast, the administration of nitrite significantly attenuated the ischaemia-induced increase in superoxide and 3-NT productions (Figure 5). This latter might be associated with the observation that the State 4 respiration was significantly increased in the NaNO_2_+I/R dogs, indicating an increase in proton leakage in the inner membrane, which results in a reduction in ROS production (Brand et al., 1999; Divakaruni and Brand, 2011).

Although we do not have direct evidence that in the protective effect of nitrite the modification of the phosphorylation system plays a major role, the fact that following the administration of the uncoupler FCCP, the decrease in ETS was similar both in the control and in the nitrite treated dogs, supports this idea. We assume that nitrite (or NO) acts on one of the components of the phosphorylation system, such as, for example, the ATP synthase, the phosphate transporter or the ADP/ATP translocator ANT. It might well be that nitrite interferes with the interaction of ATP synthase and cyclophilin D, which interaction plays a role in the formation and opening of mitochondrial permeability transition pores (MPTP), resulting in decreased ATP synthesis and increased ROS formation under conditions of I/R (Halestrap and Richardson, 2015). Moreover, the inhibition of the pore forming and opening interactions between the inner mitochondrial membrane proteins and cyclophilin D results in protection by reducing ATP loss and ROS formation (Javadov and Kuznetsov, 2013; Halestrap and Richardson, 2015). Recent evidence suggests that the cysteine 203 residue of cyclophilin D is necessary for cyclophilin D activation and subsequent MPTP opening (Nguyen et al., 2011), and that this residue undergoes protein SNO (Kohr et al., 2011). It has been suggested that in a NO-enriched environment, the formation of SNO is protective by preventing the crucial proteins from the irreversible modification of oxidation, occurring during I/R (Sun et al., 2006). It is tempting to speculate that in the delayed cardioprotective effect of nitrite, the S nitrosylation of mitochondrial proteins involved in the regulation of MPTP, plays an important role.

In summary, the results of this study confirm that the administration of sodium nitrite provides protection against the ischaemia and reperfusion-induced severe ventricular arrhythmias, 24 h later. We have now shown that this protective effect may involve, among a number of other NO-dependent effects, changes in mitochondrial morphology and function. Nitrite prevents the I/R-induced structural alterations of the mitochondria, and most probably by interfering with the phosphorylation system, inhibits the ROS producing components of the ETS and reduces the ROS formation during the early phase of reperfusion. As to whether the nitrite-induced protection attains through S-nitrosylation of proteins or one of the crucial proteins involved in the regulation of MPTP, warrants further investigations.

## Author contributions

ÁV, VD-H, and MK contributed to the conception and design of this study. The *in vivo* and *in vitro* experiments, as well as the data acquisition and analysis were performed by VD-H, MK, AP (mitochondrial respiratory measurements), and RP (TEM analysis). The drafting and revising the work was made by ÁV with the contribution of DM and LS to data interpretation. All authors participated in the manuscript revision, read and approved the submitted version.

### Conflict of interest statement

The authors declare that the research was conducted in the absence of any commercial or financial relationships that could be construed as a potential conflict of interest.

## Abbreviations

3-NT: 3-nitrotyrosine
ADP: Adenosine 5′-diphosphate
ATP: Adenosine 5′-triphosphate
CI: Mitochondrial respiratory chain complex I
CII: Mitochondrial respiratory chain complex II
CIII: Mitochondrial respiratory chain complex III
CytC: Cytochrome c
DABP: Diastolic arterial blood pressure
DHE: Dihydroethidium
ETS: Electron transport system
FCCP: Carbonyl cyanide p-(trifluoro-methoxy) phenyl-hydrazone
HR: Heart rate
IC: Ischaemic control group
I/R: Ischaemia and reperfusion
IMF: Inter-myofibrillar
LAD: Left anterior descending coronary artery
LV: Left ventricle
LVEDP: Left ventricular end-diastolic pressure
LVSP: Left ventricular systolic pressure
MABP: Mean arterial blood pressure
MPTP: Mitochondrial permeability transition pore
NO: Nitric oxide
OXPHOS: Oxidative phosphorylation
PN: Perinuclear
RCR: Respiratory control ratio
RLU: Relative luminescence unit
ROS: Reactive oxygen species
SABP: Systolic arterial blood pressure
SC: Sham-operated control group
SNO: S-nitrosylation
SSM: Sub-sarcolemmal
TEM: Transmission electronmicroscopy
VF: Ventricular fibrillation
VPBs: Ventricular premature beats
VT: Ventricular tachycardia
